# Benefit of endovascular treatment for primary versus secondary medium vessel occlusion: A multi‐center experience

**DOI:** 10.1111/cns.14687

**Published:** 2024-03-18

**Authors:** Hai‐Zhou Hu, Yong‐Gang Zhao, Xin Liu, Xian‐Hui Sun, Thanh N. Nguyen, Hui‐Sheng Chen

**Affiliations:** ^1^ Department of Neurology General Hospital of Northern Theater Command Shenyang China; ^2^ Department of Graduate School China Medical University Shenyang China; ^3^ Neurology, Radiology Boston Medical Center Boston Massachusetts USA

**Keywords:** endovascular treatment, intracranial hemorrhage, ischemic stroke, medium vessel occlusion, outcome

## Abstract

**Aims:**

This study aimed to compare the clinical outcomes and safety of endovascular treatment (EVT) in patients with primary versus secondary medium vessel occlusion (MeVO).

**Methods:**

From the endovascular treatment for acute ischemic stroke in the China registry, we collected consecutive patients with MeVO who received EVT. The primary endpoint was a good outcome, defined as a modified Rankin Scale (mRS) 0 to 2 at 90 days.

**Results:**

154 patients were enrolled in the final analysis, including 74 primary MeVO and 80 secondary MeVO. A good outcome at 90 days was achieved in 42 (56.8%) patients with primary MeVO and 33 (41.3%) patients with secondary MeVO. There was a higher probability of good outcomes in patients with the primary vs secondary MeVO (adjusted odds ratio, 2.16; 95% confidence interval, 1.04 to 4.46; *p* = 0.04). There were no significant differences in secondary and safety outcomes between MeVO groups. In the multivariable analysis, baseline ASPECTS (*p* = 0.001), final modified thrombolysis in cerebral infarction score (*p* = 0.01), and any ICH (*p* = 0.03) were significantly associated with good outcomes in primary MeVO patients, while baseline National Institutes of Health Stroke Scale (*p* = 0.002), groin puncture to recanalization time (*p* = 0.02), and early neurological improvement (*p* < 0.001) were factors associated with good outcome in secondary MeVO patients.

**Conclusion:**

In MeVO patients who received EVT, there was a higher likelihood of poor outcomes in patients with secondary versus primary MeVO.

## Graphical abstract

This study aimed to compare the clinical outcomes and safety of endovascular treatment (EVT) in patients with primary versus secondary medium vessel occlusion (MeVO). In MeVO patients who received EVT, there was higher likelihood of poor outcome in patients with secondary versus primary MeVO.

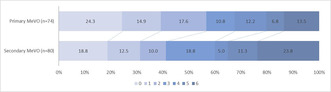

## 1 INTRODUCTION

Rapid recanalization to rescue ischemic penumbra is the cornerstone of achieving good outcomes in patients with acute ischemic stroke (AIS). Endovascular treatment (EVT) has been established as the standard care for patients with acute large vessel occlusion (LVO).^1^, ^2^ However, the benefit of EVT for other vessel occlusions such as medium vessel occlusion (MeVO) is not clear. MeVO is usually defined as occlusion of middle cerebral artery (MCA) M2‐M3 segments, anterior cerebral artery (ACA) A1‐A3 segments, and posterior cerebral artery (PCA) P1‐P3 segments, accounting for 25%–40% of acute cerebral vessel occlusions.^3^ Even in a distal occlusion location, the overall outcomes of MeVO receiving standard medical management is fair, with a third of patients not achieving functional independence at 3 months.^4^ Several retrospective studies suggest that EVT may be safe and effective in MeVO patients.^5^, ^6^, ^7^ A multicenter study found that patients with MeVO and LVO achieved similar 90‐day modified Rankin Scale (mRS), symptomatic intracranial hemorrhage (sICH) rate, and successful recanalization rate after EVT.^8^

MeVO can involve a primarily isolated thrombus, a secondary distal or new territory embolism related to EVT, intravenous thrombolysis (IVT), or spontaneous migration.^9^ Previous observational studies focused on the comparison between EVT and best medical treatment (BMT) in primary MeVO and found that there were similar functional outcomes at 90 days between patients receiving two treatments, but a higher sICH risk was observed in isolated PCA occlusion‐treated with EVT.^10^, ^11^, ^12^ Secondary MeVO caused by thrombectomy for LVO is not uncommon. One study observed that approximately 9.4% of thrombus escaped to the ACA during anterior circulation LVO thrombectomy, which was associated with increased mortality and disability.^13^ In clinical practice, many neurointerventionists choose to continue EVT when secondary MeVO occurs to seek complete recanalization, but the risks and benefits are not fully understood. Although EVT in distal occlusion may be safe and technically feasible, more thrombectomy passes may lead to blood‐brain barrier disruption, resulting in greater sICH and poor outcomes.^14^ Meanwhile, secondary MeVO may have a larger ischemic territory than primary MeVO due to previous LVO. In this context, we speculate that the clinical outcome in patients with primary MeVO may be better than that in patients with secondary MeVO. Therefore, we conducted this retrospective study to compare the effectiveness and safety of EVT in patients with primary and secondary MeVO.

## 2 METHODS

### 2.1 Patients and design

We used data from the endovascular treatment for acute ischemic stroke in China (DETECT‐China) registry, which was a retrospective, national, multi‐center, registry study evaluating the efficacy and safety of patients suffering from LVO‐AIS treated with EVT in 24 comprehensive stroke centers in China (NCT04752735). The study was approved by the ethics committee of the General Hospital of Northern Theater Command (IRB: y (2021)013) and followed the Strengthening the Reporting of Observational Studies in Epidemiology (STROBE) guidelines.^15^ Patient informed consent was waived due to the retrospective nature of this study. We enrolled adult patients with anterior circulation AIS undergoing EVT from January 2018 to January 2023. The inclusion criteria were as follows: (1) evidence of primary isolated MeVO or secondary MeVO after thrombectomy of MCA‐M1 occlusion; (2) patients who received EVT, including stent retriever, aspiration, intra‐arterial thrombolysis, rescue balloon or stenting angioplasty; and (3) mRS ≤ 2 before stroke onset. Patients with internal carotid artery occlusion, MCA‐M1 occlusion, bilateral infarction, missing procedural details, premorbid mRS > 2, or mRS data missing were excluded from our study.

### 2.2 Definition of MeVO

In this study, MeVO was confirmed by digital subtraction angiography (DSA) as occlusion of the M2, M3, A1, A2, or A3 segment, and categorized into primary and secondary etiologies.^9^, ^16^ Primary MeVO refers to an isolated distal thrombus that developed de novo without severe stenosis or occlusion in proximal large vessels.^16^, ^17^ Secondary MeVO arose from MCA‐M1 occlusion, consisting of three instances: (1) distal or new territory MeVO after EVT; (2) IVT‐induced or spontaneous thrombus migration, defined as MCA‐M1 occlusion at baseline confirmed by CT angiography, which was found as MeVO by preprocedural DSA; (3) MeVO located at the distal position of MCA‐M1 with severe stenosis or occlusion.^17^

### 2.3 Data collection

Patient demographics, medical history, National Institutes of Health Stroke Scale (NIHSS), imaging data, procedural details, and laboratory indexes were collected. The imaging data, including baseline Alberta Stroke Program Early CT Score (ASPECTS), occlusion site, and postprocedural intracranial hemorrhage (ICH), were evaluated by a neuroradiologist. If the CT image was not of adequate quality to assign an ASPECTS or if there was a lack of CT imaging at admission (due to transfer patients), it was recorded as missing. The preprocedural collateral status and postprocedural mTICI grading were evaluated by a neurologist during the procedure and recorded in the medical record. If there were no relevant records, the data were obtained by a neurologist who read these images. We recorded procedure complications, including target vessel perforation, dissection, and vasospasm. The 90‐day mRS score was evaluated through telephone interviews or in‐person follow‐up.

### 2.4 Outcomes

The primary endpoint was a good outcome, defined as mRS 0–2 at 90 days. Secondary endpoints included the proportion of patients with 90‐day mRS 0–1 and 0–3, 90‐day mRS distribution, early neurological improvement (ENI), and change in NIHSS score at 24 h, 48 h, 7 days, and at discharge. Safety endpoints were the proportion of sICH within 24 h, the proportion of any ICH within 24 h, and all‐cause mortality at 7 days and 90 days. ENI was defined as a decrease of more than 4 points in NIHSS score from baseline at 24 h,^18^ and sICH was defined as an increase of more than 4 points in NIHSS score caused by ICH.^19^

### 2.5 Statistical analysis

SPSS V.25.0 software was used to conduct the statistical analyses. Shapiro–Wilk test was used to assess data distribution. Continuous variables were presented as mean ± SD or median (IQR), and compared with *t*‐test or Mann–Whitney *U*‐test depending on their normality. Categorical variables were described using numbers (percentage) and compared between groups via Pearson's *χ* ^2^ test or Fisher's exact test as appropriate. To compare the functional outcomes of primary MeVO and secondary MeVO, we performed binary and ordinal logistic regression models to evaluate the 90‐day mRS and its distribution, sICH, and any ICH. A generalized linear model was used to analyze the change in NIHSS score, and Cox regression analysis was used to analyze all‐cause mortality. We adjusted for confounding factors a priori that are known to affect patient outcomes, such as age, gender, baseline NIHSS, pretreatment with IVT, pre‐stroke mRS, onset to puncture time (OTP), groin puncture to recanalization time (GTR), preprocedural collateral, and final mTICI. Next, the MeVO population was divided into the good outcome and poor outcome groups. Univariable logistic regression analysis was conducted. Variables with *p* < 0.05 were identified as potential predictors. The variance inflation factor (VIF) was used to evaluate their multicollinearity (VIF < 3 was considered non‐collinearity), and qualified variables were included in a stepwise logistic regression model to analyze independent factors associated with a 90‐day good outcome. We also performed a subgroup analysis regarding outcomes of different M2 occlusion types (dominant or nondominant) in the primary versus secondary group with interaction test, and adjusted for age, baseline NIHSS, pretreatment with IVT, and final mTICI. All statistical tests were two‐sided, with *p* < 0.05 considered statistically significant.

## 3 RESULTS

**FIGURE 1:**
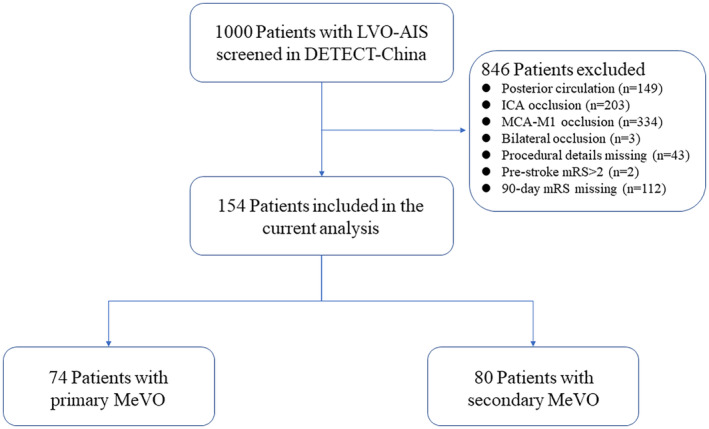
Study flowchart of patient enrollment. AIS, acute ischemic stroke; ICA, internal carotid artery; LVO, large vessel occlusion; MCA‐M1, middle cerebral artery M1 segment; MeVO, medium vessel occlusion; mRS, modified Rankin Scale.

Of 1000 patients in DETECT, 846 patients were excluded, including 149 posterior circulation infarctions, 203 internal carotid artery occlusions, 334 MCA‐M1 occlusions, 3 bilateral occlusion, 43 without procedural details, 2 pre‐stroke mRS > 2, and 112 mRS data missing (consisted of 107 MCA‐M1 occlusion, 4 primary MeVO, and 1 secondary MeVO). A total of 154 patients were enrolled in this analysis, including 74 primary MeVO and 80 secondary MeVO (Figure 1). The baseline characteristics and outcomes are shown in Table 1. In the entire cohort, the median age was 66 (IQR, 56–73) years, with 97 (63.0%) male patients. The median baseline NIHSS score was 14 (IQR, 11–17), and 51 (33.1%) patients received IVT. Median onset to recanalization time was 394 (IQR, 308–520), and 118 (76.6%) patients achieved successful recanalization. Two patients (1.3%) underwent procedural complications. The occlusion site of MeVO was mostly the MCA M2 segment (88.3%). Compared to patients with secondary MeVO, the primary MeVO group had fewer smokers (32.4% vs. 51.2%, *p* = 0.02), fewer preprocedural leptomeningeal collaterals (LMC) (23.0% vs. 45.0%, *p* = 0.004), less use of stent retriever (70.3% vs. 95.0%, *p* < 0.001) and aspiration (18.9% vs. 45.0%, *p* = 0.001), and lower number of MT passes (1 [0–2] vs. 2 [2, 3], *p* < 0.001). There was a trend of more male patients (70.0% vs. 55.4%, *p* = 0.06), longer GTR (92 [60–134] vs. 76 [49–110], *p* = 0.05), and fewer MCA‐M2 occlusion (83.8% vs. 93.2%, *p* = 0.07) in secondary MeVO group. For the primary outcome of mRS 0–2 at 90 days, this was achieved in 42 (56.8%) patients in the primary MeVO group and 33 (41.3%) patients in the secondary MeVO group (Table 1).

**TABLE 1:** Baseline characteristics and outcome of patients with primary and secondary MeVO.

Variables	Total (*n* = 154)	Primary MeVO (*n* = 74)	Secondary MeVO (*n* = 80)	*p* Value
Age, year, median (IQR)	66 (56–73)	67 (60–74)	65 (53–73)	0.101
Men, *n* (%)	97 (63.0)	41 (55.4)	56 (70.0)	0.061
Medical history, *n* (%)				
Hypertension	89 (57.8)	47 (63.5)	42 (52.5)	0.167
Diabetes mellitus	37 (24.0)	19 (25.7)	18 (22.5)	0.645
Hyperlipidemia^a^	34 (23.6)	13 (19.1)	21 (27.6)	0.230
Coronary heart disease	34 (22.1)	17 (23.0)	17 (21.3)	0.797
Atrial fibrillation	74 (48.1)	34 (45.9)	40 (50.0)	0.615
Prior stroke	48 (31.2)	25 (33.8)	23 (28.7)	0.500
Smoking (recent or current)	65 (42.2)	24 (32.4)	41 (51.2)	**0.018**
Drinking (recent or current)	66 (42.9)	27 (36.5)	39 (48.8)	0.124
Pre‐stroke mRS, *n* (%)				
0	131 (85.1)	63 (85.1)	68 (85.0)	0.984
1	16 (10.4)	7 (9.5)	9 (11.2)	
2	7 (4.5)	4 (5.4)	3 (3.8)	
Baseline NIHSS, median (IQR)	14 (11–17)	13 (11–17)	14 (11–17)	0.198
Baseline ASPECTS, median (IQR)^b^	9 (8–10)	9 (8–10)	9 (8–10)	0.888
Left‐sided infarction, *n* (%)	78 (50.6)	39 (52.7)	39 (48.8)	0.624
Pre‐treatment with IVT, *n* (%)	51 (33.1)	24 (32.4)	27 (33.8)	0.862
Duration, min, median (IQR)				
OTP	303 (230–425)	299 (230–444)	320 (229–405)	0.759
GTR	83 (55–120)	76 (49–110)	92 (60–134)	0.051
OTR	394 (308–520)	394 (298–541)	395 (309–509)	0.688
LMC, *n* (%)	53 (34.4)	17 (23.0)	36 (45.0)	**0.004**
EVT, *n* (%)				
Stent retriever	128 (83.1)	52 (70.3)	76 (95.0)	**<0.001**
Aspiration	50 (32.5)	14 (18.9)	36 (45.0)	**0.001**
IAT	41 (26.6)	24 (32.4)	17 (21.3)	0.117
Rescue balloon angioplasty	11 (7.1)	3 (4.1)	8 (10.0)	0.263
Rescue stenting angioplasty	7 (4.5)	3 (4.1)	4 (5.0)	1.000
MT passes, *n*, median (IQR)				
MeVO	1 (0–1)	1 (0–2)	1 (0–1)	0.763
MCA‐M1	0 (0–1)	NA	1 (1–2)	NA
Total	2 (1–2)	1 (0–2)	2 (2–3)	**<0.001**
Final mTICI 2b‐3, *n* (%)	118 (76.6)	55 (74.3)	63 (78.8)	0.517
Procedural complications, *n* (%)	2 (1.3)	0 (0)	2 (2.5)	0.497
Cause of vessel occlusion, *n* (%)				
Atherosclerosis	56 (36.4)	27 (36.5)	29 (36.3)	0.642
Cardioembolism	61 (39.6)	27 (36.5)	34 (42.5)	
Other or unknown etiology	37 (34.0)	20 (27.0)	17 (21.3)	
Occlusion location, *n* (%)				
MCA‐M2	136 (88.3)	69 (93.2)	67 (83.8)	0.067
Dominant‐M2	63 (40.9)	36 (52.2)	27 (38.8)	0.060
Nondominant‐M2	73 (47.4)	33 (47.8)	40 (61.2)	0.502
MCA‐M3	12 (7.8)	3 (4.1)	9 (11.3)	0.173
ACA‐A1	5 (3.2)	4 (5.4)	1 (1.3)	0.196
ACA‐A2	7 (4.5)	2 (2.7)	5 (6.3)	0.609
Primary outcome				
90‐day mRS 0–2, *n* (%)	75 (48.7)	42 (56.8)	33 (41.3)	0.054
Secondary outcomes				
90‐day mRS, median (IQR)	3 (1–5)	2 (1–4)	3 (1–5)	0.084
90‐day mRS 0–1, *n* (%)	54 (35.1)	29 (39.2)	25 (31.3)	0.302
90‐day mRS 0–3, *n* (%)	98 (63.6)	50 (67.6)	48 (60.0)	0.329
ENI, *n* (%)	52 (33.8)	27 (36.5)	25 (31.3)	0.492
Change in NIHSS, median (IQR)				
At 24 h^c^	1.5 (0 to 5)	2 (0 to 5)	1 (0 to 5)	0.237
At 48 h^d^	2 (0 to 6)	2 (0 to 6)	2 (−1.25 to 7)	0.255
At 7 days^e^	5 (1 to 8)	5 (2 to 9)	4.5 (0.25 to 8)	0.179
At discharge^d^	5 (0.5 to 9.5)	6 (1 to 10)	4.5 (0 to 8.25)	0.126
Safety outcomes				
SICH at 24 h, *n* (%)	21 (13.6)	6 (8.1)	15 (19.0)	0.055
Any ICH at 24 h, *n* (%)	52 (33.8)	19 (25.7)	33 (41.8)	**0.041**
Mortality within 7 days, *n* (%)	17 (11.1)	6 (8.1)	11 (13.8)	0.264
Mortality within 90 days, *n* (%)	29 (18.8)	10 (13.5)	19 (23.8)	0.105

*Note*: Bold values indicate statistical significance.

Abbreviations: ACA, anterior cerebral artery; ASPECTS, Alberta Stroke Program Early CT Score; ENI, early neurological improvement; EVT, endovascular treatment; GTR, groin puncture to recanalization time; IAT, Intra‐arterial thrombolysis; ICH, intracranial hemorrhage; IVT, intravenous thrombolysis; LMC, leptomeningeal collaterals; MCA, middle cerebral artery; MeVO, medium vessel occlusion; mRS, modified Rankin Scale; MT, mechanical thrombectomy; mTICI, modified thrombolysis in cerebral infarction score; NIHSS, National Institute of Health Stroke Scale; OTP, onset to puncture time; OTR, onset to recanalization time; SICH, symptomatic intracranial hemorrhage.

^a^ Ten missing data.

^b^ Eight missing data.

^c^ Two missing data.

^d^ Nine missing data.

^e^ Nineteen missing data.

**FIGURE 2:**
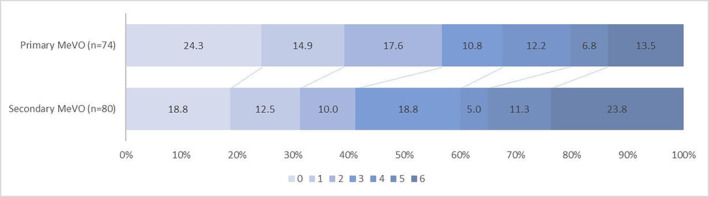
90‐day modified Rankin Scale (mRS) distribution. MeVO, medium vessel occlusion.

The comparison of clinical and safety outcomes between the two groups is shown in Table 2 and Figure 2. In the adjusted model, the proportion of patients with 90‐day mRS 0–2 was higher in the primary MeVO compared with the secondary MeVO group (56.8% vs. 41.3%, adjusted odds ratio [aOR], 2.16; 95% confidence interval [CI], 1.04 to 4.46; *p* = 0.04). There was no difference in secondary outcomes between primary and secondary MeVO groups. For safety outcomes, there was a trend that primary MeVO had a lower risk of sICH (8.1% vs. 19.0%, aOR, 0.32; 95% CI, 0.10 to 1.01; *p* = 0.05) compared to secondary MeVO.

**TABLE 2:** Efficacy and safety outcome in patients with primary and secondary MeVO.

Outcomes Primary vs. secondary	Effect metric	Unadjusted model		Adjusted model^a^	
		Difference (95% CI)	*p*	Difference (95% CI)	*p*
Primary outcome					
90‐day mRS 0–2	OR	1.869 (0.986 to 3.545)	0.055	2.158 (1.044 to 4.464)	**0.038**
Secondary outcomes					
90‐day mRS 0–1	OR	1.418 (0.730 to 2.755)	0.303	1.529 (0.738 to 3.170)	0.254
90‐day mRS 0–3	OR	1.389 (0.717 to 2.690)	0.330	1.619 (0.759 to 3.452)	0.212
90‐day mRS^b^	cOR	1.672 (0.959 to 2.915)	0.070	1.710 (0.961 to 3.040)	0.068
ENI	OR	1.264 (0.647 to 2.467)	0.493	1.487 (0.731 to 3.022)	0.273
Change in NIHSS^c^					
At 24 h^e^	GMR	1.062 (0.976 to 1.156)	0.160	1.072 (0.986 to 1.165)	0.103
At 48 h^f^	GMR	1.055 (0.956 to 1.165)	0.287	1.062 (0.962 to 1.172)	0.233
At 7 days^g^	GMR	1.069 (0.947 to 1.207)	0.283	1.093 (0.971 to 1.231)	0.140
At discharge^f^	GMR	1.087 (0.960 to 1.231)	0.186	1.097 (0.972 to 1.239)	0.133
Safety outcomes					
SICH at 24 h	OR	0.382 (0.140 to 1.046)	0.061	0.324 (0.104 to 1.005)	0.051
Any ICH at 24 h	OR	0.492 (0.248 to 0.977)	**0.043**	0.554 (0.270 to 1.137)	0.107
Mortality within 7 days^d^	HR	0.575 (0.213 to 1.555)	0.275	0.619 (0.220 to 1.746)	0.365
Mortality within 90 days^d^	HR	0.539 (0.251 to 1.160)	0.114	0.525 (0.236 to 1.166)	0.113

*Note*: Bold values indicate statistical significance.

Abbreviations: CI, confidence interval; cOR, common odds ratio; ENI, early neurological improvement; GMR, geometric mean ratio; HR, hazard ratio; ICH, intracranial hemorrhage; MeVO, medium vessel occlusion; mRS, modified Rankin Scale; NIHSS, National Institute of Health Stroke Scale; OR, odds ratio; SICH, symptomatic intracranial hemorrhage.

^a^ Adjusted for key prognostic covariates (age, sex, pre‐treatment with IVT, pre‐stroke mRS, baseline NIHSS, OTP, GTR, LMC and final mTICI).

^b^ Calculated with ordinal regression analysis.

^c^ The log (NIHSS + 1) was analyzed using a generalized linear model.

^d^ Calculated with Cox regression model.

^e^ Two missing data.

^f^ Nine missing data.

^g^ Nineteen missing data.

Compared to MeVO patients with poor outcome, those who achieved good outcome were younger (64 [54–69] vs. 68 [59–76], *p* = 0.009), had lower baseline NIHSS (12 [10–15] vs. 16 [13–19], *p* < 0.001), higher baseline ASPECTS (9 [9, 10] vs. 8 [8, 9], *p* < 0.001), lower baseline neutrophil‐to‐lymphocyte ratio (5.1 [3.3–7.4] vs. 7.4 [4.2–10.8], *p* = 0.002), fewer MT passes times (1 [1, 2] vs. 2 [1–3], *p* = 0.007), shorter GTR (60 [47–100] vs. 100 [64–144], *p* < 0.001), higher successful recanalization rate (89.3% vs. 64.6%, *p* = 0.001), higher proportion of ENI (53.3% vs. 15.2%, *p* < 0.001), and lower incidence of ICH (18.7% vs. 48.7%, *p* < 0.001) or sICH (4.0% vs. 23.1%, *p* = 0.002) (Table 3). In the multivariable analysis, baseline ASPECTS (aOR, 3.93; CI, 1.71 to 9.03; *p* = 0.001), final mTICI 2b‐3 (aOR, 6.80; CI, 1.30 to 31.10; *p* = 0.01), and any ICH (aOR, 0.22; CI, 0.05 to 0.87; *p* = 0.03) were significantly associated with good outcome in primary MeVO (Table 4). In secondary MeVO, baseline NIHSS (aOR, 0.77; CI, 0.65 to 0.91; *p* = 0.002), GTR (aOR, 0.98; CI, 0.97 to 1.00; *p* = 0.02), and ENI (aOR, 13.08; CI, 3.22 to 53.13; *p* < 0.001) were independent predictors of good outcome (Table 4).

**TABLE 3:** Univariate and multivariable analyses regarding good and poor outcomes of all MeVO patients.

Variables^a^	Good outcome	Poor outcome	Univariate analysis		Multivariable analysis^b^	
	(*n* = 75)	(*n* = 79)	Crude OR (95% CI)	*p*	Adjusted OR (95% CI)	*p*
Age, year, median (IQR)	64 (54–69)	68 (59–76)	0.967 (0.942–0.992)	0.009	0.934 (0.890–0.980)	0.005
Baseline NIHSS, median (IQR)	12 (10–15)	16 (13–19)	0.833 (0.764–0.909)	<0.001	0.866 (0.766–0.979)	0.022
Baseline ASPECTS, median (IQR)^c^	9 (9–10)	8 (8–9)	2.497 (1.637–3.810)	<0.001	2.316 (1.286–4.171)	0.005
Baseline NLR, median (IQR)^d^	5.1 (3.3–7.4)	7.4 (4.2–10.8)	0.895 (0.834–0.961)	0.002	0.886 (0.805–0.975)	0.013
GTR, min, median (IQR)	60 (47–100)	100 (64–144)	0.984 (0.976–0.992)	<0.001		0.089
MT passes, *n*, median (IQR)	1 (1–2)	2 (1–3)	0.689 (0.525–0.905)	0.007	0.577 (0.344–0.996)	0.036
Final mTICI 2b‐3, *n* (%)	67 (89.3)	51 (64.6)	4.598 (1.934–10.932)	0.001	4.407 (1.169–16.613)	0.028
Early neurological improvement, *n* (%)	40 (53.3)	12 (15.2)	6.381 (2.973–13.695)	<0.001	5.665 (1.883–17.040)	0.002
Symptomatic intracranial hemorrhage, *n* (%)^d^	3 (4.0)	18 (23.1)	0.141 (0.040–0.502)	0.002		0.525
Any intracranial hemorrhage, *n* (%)^d^	14 (18.7)	38 (48.7)	0.248 (0.119–0.514)	<0.001	0.175 (0.052–0.592)	0.005

Abbreviations: ASPECTS, Alberta Stroke Program Early CT Score; GTR, groin puncture to recanalization time; MeVO, medium vessel occlusion; MT, mechanical thrombectomy; mTICI, modified thrombolysis in cerebral infarction score; NIHSS, National Institute of Health Stroke Scale; NLR, neutrophil‐to‐lymphocyte ratio.

^a^ Only variables with *p* < 0.05 in univariable logistic regression are listed.

^b^ Calculated with stepwise logistic regression model.

^c^ Eight missing data.

^d^ One missing data.

**TABLE 4:** Univariable and multivariable analyses regarding good and poor outcomes in patients with medium vessel occlusion (MeVO).

Variables^a^	Good outcome	Poor outcome	Univariate analysis		Multivariable analysis^b^	
			Crude OR (95% CI)	*p*	Adjusted OR (95% CI)	*p*
Primary MeVO	*n* = 42	*n* = 32				
Baseline NIHSS, median (IQR)	12 (10–15)	15 (12–19)	0.854 (0.755–0.965)	0.012		0.171
Baseline ASPECTS, median (IQR)^c^	9 (9–10)	8 (8–9)	4.462 (2.040–9.760)	<0.001	3.930 (1.710–9.034)	**0.001**
Baseline NLR, median (IQR)	4.8 (3.3–6.2)	7.2 (4.2–10.3)	0.839 (0.724–0.973)	0.020		0.070
GTR, min, median (IQR)	60 (42–105)	89 (60–140)	0.988 (0.978–0.999)	0.028		0.475
Final mTICI 2b‐3, *n* (%)	37 (88.1)	18 (56.3)	5.756 (1.793–18.473)	0.003	6.801 (1.303–31.098)	**0.013**
Early neurological improvement, *n* (%)	21 (50.0)	6 (18.8)	4.333 (1.480–12.686)	0.007		0.086
Any intracranial hemorrhage, *n* (%)	6 (14.3)	13 (40.6)	0.244 (0.080–0.743)	0.013	0.216 (0.054–0.870)	**0.031**
Secondary MeVO	*n* = 33	*n* = 47				
Age, year, median (IQR)	61 (52–67)	69 (54–74)	0.965 (0.931–0.999)	0.049		0.145
Baseline NIHSS, median (IQR)	12 (10–15)	16 (13–19)	0.818 (0.721–0.929)	0.002	0.769 (0.651–0.909)	**0.002**
Baseline ASPECTS, median (IQR)^d^	9 (9–10)	8 (8–9)	1.877 (1.104–3.191)	0.020		0.134
GTR, min, median (IQR)	61 (50–96)	110 (75–150)	0.981 (0.969–0.993)	0.002	0.984 (0.970–0.998)	**0.022**
Early neurological improvement, *n* (%)	19 (57.6)	6 (12.8)	9.274 (3.086–27.867)	<0.001	13.081 (3.221–53.127)	**<0.001**
Any intracranial hemorrhage, *n* (%)^e^	8 (24.2)	25 (54.3)	0.269 (0.100–0.720)	0.009		0.150

*Note*: Bold values indicate statistical significance in multivariable analysis.

Abbreviations: ASPECTS, Alberta Stroke Program Early CT Score; GTR, groin puncture to recanalization time; mTICI, modified thrombolysis in cerebral infarction score; NIHSS, National Institute of Health Stroke Scale; NLR, neutrophil‐to‐lymphocyte ratio.

^a^ Only variables with *p* < 0.05 in univariable logistic regression are listed.

^b^ Calculated with stepwise logistic regression model.

^c^ Six missing data.

^d^ Two missing data.

^e^ One missing data.

Tables S1 and S2 show the comparison of characteristics and outcomes in primary versus secondary M2 occlusion according to dominant and nondominant types. The primary group had a lower risk of ICH than the secondary group in nondominant‐M2 occlusion (aOR, 0.26; CI, 0.09 to 0.89; adjusted *p* = 0.03). No significant difference was found in other outcomes (all *p* > 0.05), and there was no interaction effect of dominant and nondominant types in primary versus secondary M2 occlusion for all outcomes (all *p*_interaction_ > 0.05).

## 4 DISCUSSION

In this study, we found that patients with primary MeVO had a higher probability of 90‐day functional independence compared to those secondary MeVO. In addition, baseline ASPECTS, final mTICI 2b‐3, and any ICH were significantly associated with good outcomes in primary MeVO, while baseline NIHSS, GTR, and ENI were factors or metrics associated with good outcomes in secondary MeVO. Different type of M2 occlusion was not associated with primary versus secondary groups for all outcomes.

The clinical outcomes and safety of EVT for MeVO patients are not fully understood. IVT remains the first‐line treatment recommended by international guidelines.^1^ However, a post hoc analysis of INTERRSeCT and PRoveIT showed that only half of MeVO patients receiving the best medical treatment achieved 90‐day mRS 0–1, with an early recanalization rate of less than 50% after IVT.^4^ It is known that early recanalization is highly related to good prognosis, suggesting that medical treatment alone may not be the best option for MeVO patients. Across physician surveys, a growing number of interventionists would consider EVT for MeVO patients, particularly if they had not received preceding IVT.^16^, ^20^

When a thrombus moves to a distal location, irreversible ischemic injury may have occurred in the blood supply area of the proximal occluded large vessels. Thus, a worse prognosis is expected to be observed in secondary MeVO compared with primary MeVO. The hypothesis was partially confirmed in this study because a greater proportion of primary MeVO patients achieved good outcomes than secondary MeVO patients. However, there was no difference in secondary outcomes between the two MeVO groups. This may be related to the relatively small sample size and heterogeneity of patients, for example, the high baseline NIHSS in the primary MeVO group. This further suggests a selection bias that primary MeVO patients with severe neurological deficits were more likely to receive EVT.^21^ In addition, most confirmation of MeVO in this study depended on DSA; however, there was a possibility of large vessel thrombus migrating to a more distal vessel,^17^ resulting in the fact that some patients with secondary MeVO might have been mixed in the primary MeVO group. These factors may affect the outcome of primary MeVO.

ICH is a common complication of EVT.^22^ The small diameter, fragile wall, and tortuous access pathways of medium‐sized vessels undoubtedly increase the technical difficulty of EVT. In our study, the incidence of ICH in the MeVO group was high (34.0%), and was more significant in the secondary MeVO, with up to 41.8% rate of ICH (of which, 19.0% were sICH). Given the poor performance of non‐contrast CT to detect small infarcts, it was probable that EVT was performed for MeVO in which the blood‐supply area was already infarcted, which may partially explain the higher ICH rate. Furthermore, more severe endothelium injury^23^ and mechanical blood‐brain barrier disruption^14^ caused by frequent MT passes could be another reason for the higher incidence of ICH in secondary MeVO. Interestingly, compared with LVO patients in the HERMES collaboration,^24^ we found that the patients with secondary MeVO had a higher incidence of atrial fibrillation (50% vs. 33%). Of note, cardiogenic emboli may be a risk factor for ICH due to secondary embolism during EVT.^25^ All patients who died in the primary MeVO group had sICH, with the mortality event occurring within 7 days. This suggests that ICH occurring during the procedure may be at high risk of mortality in patients with MeVO. These results suggest that interventionists should balance the benefits and risks to carefully choose treatment strategies. Some studies found that Tigetriever13 (a low‐profile stent retriever), MIVI Q catheters (a novel aspiration catheter), and the blind exchange/mini‐pinning (BEMP) technique may be safe and effective for MeVO.^26^, ^27^, ^28^, ^29^ In addition, general anesthesia can reduce patient movement and agitation, making it easier for the guide wire or catheter to reach the distal vessels, which may improve the safety of EVT for MeVO.^9^ Nevertheless, for secondary MeVO, switching anesthesia methods during the procedure would not be the choice of most physicians.^30^

The baseline ASPECTS has been a strong predictor of functional outcome in AIS,^31^ which was also observed in primary MeVO in this study. It is widely accepted that successful recanalization is associated with good outcomes after EVT.^32^ However, the rate of final mTICI 2b‐3 in primary MeVO in our study was lower than that reported by Sun et al.^8^ (74.3% vs. 89.8%), which might be attributed to the heterogeneity of clot composition, EVT equipment, and technology in various centers. In addition, any ICH was found as an important predictor of poor outcomes for primary MeVO, indicating that physician proceduralists should be cautious when performing EVT to minimize injury to distal vessels to avoid the occurrence of ICH. Target thrombus can escape to the downstream or new territory during EVT, resulting in secondary MeVO,^9^ which was associated with frequent MT passes, prolonged GTR, high sICH rate, and poor outcome.^33^ Distal emboli occurred in 19.3% of MCA patients in our study, which was higher than that reported in previous studies.^34^ It is noteworthy that secondary MeVO had poorer outcomes compared with LVO,^13^ suggesting the need for more effective EVT methods to achieve complete recanalization, such as intra‐arterial thrombolysis during EVT.^35^, ^36^

The presence of LMC varied by the location of arterial occlusion and was associated with good outcomes.^37^, ^38^, ^39^ In this study, the proportion of LMC in patients with primary MeVO was significantly lower than that in patients with secondary MeVO (23% vs. 45%). We speculate that it may be related to the lower pressure gradients at the distal position^40^ and the lower proportion of atherosclerotic‐related stroke in distal arterial territories.^41^ The difference in collateral circulation compensation between MeVO and LVO needs further study.

Several limitations should be clarified. First, there were selection bias, information bias, and confounding bias due to the retrospective design of this study. To reduce bias, we adjusted for confounding factors but acknowledged there may be residual confounding. Second, as this was a multicenter study, there was an inevitable heterogeneity of treatment protocols, operator experience, and technique that could have affected the results. Third, the majority of MeVO in this study were M2 occlusion, so the results cannot be extrapolated to other MeVO groups. The high proportion of M2 occlusion could be because patients with MeVO and more severe symptoms were more likely to undergo EVT in real‐world clinical practice. In addition, dominant M2 occlusion is more like LVO, even if we did not find the interaction effect of dominant versus nondominant M2, it may be due to the restricted number of each group. Finally, the relatively small sample size of MeVO limited our ability to conduct subgroup analyses such as EVT methods. The benefits of different EVT strategies for MeVO patients are not yet clear and may be the direction of future research.

## 5 CONCLUSION

In MeVO patients who received EVT, patients with secondary MeVO had a higher probability of poor outcomes compared to those with primary MeVO.

## AUTHOR CONTRIBUTIONS

H.‐Z.H., Y.‐G.Z., X.L. and X.‐H.S. contributed to the acquisition and analysis of data. H.‐Z.H. and Y.‐G.Z. drafted the first manuscript. T.N.N. critically revised the manuscript. H.‐S.C. designed the study and critically revised the manuscript.

## FUNDING INFORMATION

None.

## CONFLICT OF INTEREST STATEMENT

None.

## Supporting information


Appendix S1.

Appendix S2.

## Data Availability

Data are available upon reasonable request to the corresponding author.
