# Supplementary material for: Benefit of endovascular treatment for primary versus secondary medium vessel occlusion: A multi‐center experience
Source: CNS Neurosci Ther. 2024 Mar 18;30(3):e14687. doi: 10.1111/cns.14687 (PMC10945881; doi:10.1111/cns.14687)
Supplement: Supplementary file 2 — Appendix S2. [file CNS-30-e14687-s001.docx]

Table S1. Baseline characteristics and outcome of patients with primary versus secondary M2 occlusion by dominant/nondominant types

| Variables | Dominant M2 occlusion (n=63) | | P value | Nondominant M2 occlusion (n=73) | | P value |
| --- | --- | --- | --- | --- | --- | --- |
|  | Primary (n=36) | Secondary (n=27) |  | Primary (n=33) | Secondary (n=40) |  |
| Age, year, median (IQR) | 67 (62-77) | 63 (54-69) | 0.191 | 68 (60-74) | 66 (51-73) | 0.270 |
| Men, n (%) | 20 (55.6) | 19 (70.4) | 0.231 | 19 (57.6) | 28 (70.0) | 0.270 |
| Medical history, n (%) |  |  |  |  |  |  |
| Hypertension | 20 (55.6) | 13 (48.1) | 0.560 | 24 (72.7) | 21 (52.5) | 0.077 |
| Diabetes mellitus | 8 (22.2) | 6 (22.2) | 1.000 | 8 (24.2) | 8 (20.0) | 0.663 |
| Hyperlipidemia* | 3 (8.3) | 8 (32.0) | **0.043** | 9 (32.1) | 12 (30.8) | 0.905 |
| Coronary heart disease | 9 (25.0) | 5 (18.5) | 0.540 | 8 (24.2) | 8 (20.0) | 0.663 |
| Atrial fibrillation | 13 (36.1) | 14 (51.9) | 0.212 | 18 (54.5) | 23 (57.5) | 0.800 |
| Prior stroke | 13 (36.1) | 6 (22.2) | 0.235 | 11 (33.3) | 14 (35.0) | 0.881 |
| Smoking (recent or current) | 10 (27.8) | 16 (59.3) | **0.012** | 13 (39.4) | 21 (52.5) | 0.264 |
| Drinking (recent or current) | 14 (38.9) | 16 (59.3) | 0.109 | 12 (36.4) | 19 (47.5) | 0.338 |
| Pre-stroke mRS, n (%) |  |  | 0.364 |  |  | 0.521 |
| 0 | 29 (80.6) | 24 (88.9) |  | 29 (87.9) | 33 (82.5) |  |
| 1 | 4 (11.1) | 2 (7.4) |  | 3 (9.1) | 5 (12.5) |  |
| 2 | 3 (8.3) | 1 (3.7) |  | 1 (3.0) | 2 (5.0) |  |
| Baseline NIHSS, median (IQR) | 12 (10-16) | 16 (11-18) | 0.115 | 14 (12-17) | 14 (11-17) | 0.956 |
| Baseline ASPECTS, median (IQR)※ | 9 (8-9) | 9 (8-10) | 0.159 | 9 (8-10) | 9 (8-10) | 0.754 |
| Left-sided infarction, n (%) | 18 (50.0) | 16 (59.3) | 0.466 | 19 (57.6) | 17 (42.5) | 0.200 |
| Pre-treatment with IVT, n (%) | 13 (36.1) | 15 (55.6) | 0.124 | 10 (30.3) | 10 (25.0) | 0.613 |
| Duration, min, median (IQR) |  |  |  |  |  |  |
| OTP | 343 (245-499) | 320 (240-382) | 0.090 | 286 (204-408) | 323 (219-427) | 0.468 |
| GTR | 84 (53-124) | 100 (58-144) | 0.345 | 60 (43-90) | 88 (61-120) | **0.013** |
| OTR | 465 (320-608) | 420 (323-482) | 0.227 | 340 (270-458) | 410 (311-518) | 0.129 |
| LMC, n (%) | 9 (25.0) | 10 (37.0) | 0.303 | 7 (21.2) | 21 (52.5) | **0.006** |
| EVT, n (%) |  |  |  |  |  |  |
| Stent retriever | 25 (69.4) | 26 (96.3) | **0.018** | 24 (72.7) | 39 (97.5) | **0.006** |
| Aspiration | 9 (25.0) | 16 (59.3) | **0.006** | 4 (12.1) | 17 (42.5) | **0.009** |
| IAT | 11 (30.6) | 6 (22.2) | 0.461 | 11 (33.3) | 7 (17.5) | 0.118 |
| Rescue balloon angioplasty | 3 (8.3) | 1 (3.7) | 0.629 | 0 (0) | 6 (15.0) | **0.029** |
| Rescue stenting angioplasty | 3 (8.3) | 2 (7.4) | 1.000 | 0 (0) | 2 (5.0) | 0.498 |
| MT passes, n, median (IQR) |  |  |  |  |  |  |
| MeVO | 1 (0-2) | 1 (1-1) | 0.952 | 1 (0-2) | 1 (1-2) | 0.739 |
| MCAO | NA | 1 (1-2) |  | NA | 1 (1-2) |  |
| Total | 1 (0-2) | 2 (2-3) | **<0.001** | 1 (0-2) | 2 (2-3) | **<0.001** |
| Final mTICI 2b-3, n (%) | 28 (77.8) | 22 (81.5) | 0.719 | 26 (78.8) | 31 (77.5) | 0.895 |
| Procedural complications, n (%) | 0 (0) | 0 (0) | NA | 0 (0) | 2 (5.0) | 0.498 |
| Cause of vessel occlusion, n (%) |  |  | 0.399 |  |  | 0.411 |
| Atherosclerosis | 18 (50.0) | 10 (37.0) |  | 8 (24.2) | 13 (32.5) |  |
| Cardioembolism | 9 (25.0) | 11 (40.7) |  | 15 (45.5) | 20 (50.0) |  |
| Other or unknown etiology | 9 (25.0) | 6 (22.2) |  | 10 (30.3) | 7 (17.5) |  |
| Primary outcome |  |  |  |  |  |  |
| 90-day mRS 0-2, n (%) | 18 (50.0) | 9 (33.3) | 0.186 | 21 (63.6) | 20 (50.0) | 0.243 |
| Secondary outcomes |  |  |  |  |  |  |
| 90-day mRS, median (IQR) | 3 (1-4) | 3 (1-5) | 0.211 | 2 (0-4) | 2 (0-4) | 0.349 |
| 90-day mRS 0-1, n (%) | 13 (36.1) | 8 (29.6) | 0.589 | 14 (42.4) | 15 (37.5) | 0.669 |
| 90-day mRS 0-3, n (%) | 22 (61.1) | 14 (51.9) | 0.462 | 25 (75.8) | 27 (67.5) | 0.438 |
| ENI, n (%) | 14 (38.9) | 8 (29.6) | 0.446 | 12 (36.4) | 14 (35.0) | 0.904 |
| Change in NIHSS, median (IQR) |  |  |  |  |  |  |
| at 24 hours† | 2 (0 to 4.75) | 0 (0 to 5) | 0.210 | 2 (0 to 5) | 1 (0 to 5.75) | 0.456 |
| at 48 hours* | 3 (0 to 5) | 2 (-1 to 7) | 0.230 | 2.5 (0 to 8) | 2 (-2 to 7) | 0.326 |
| at 7 days‡ | 5 (2.75 to 7.25) | 4 (0 to 7) | 0.175 | 6 (1.5 to 10.5) | 5 (1.75 to 9) | 0.567 |
| at discharge§ | 6 (2 to 9) | 2 (0 to 7) | 0.059 | 6 (1 to 11) | 5.5 (2 to 9.75) | 0.584 |
| Safety outcomes |  |  |  |  |  |  |
| SICH at 24 hours, n (%) | 2 (5.6) | 1 (3.7) | 1.000 | 3 (9.1) | 11 (27.5) | 0.091 |
| Any ICH at 24 hours, n (%) | 10 (27.8) | 8 (29.6) | 0.872 | 7 (21.2) | 20 (50.0) | **0.011** |
| Mortality within 7 days, n (%) | 2 (5.6) | 5 (18.5) | 0.224 | 3 (9.1) | 5 (12.5) | 0.930 |
| Mortality within 90 days, n (%) | 5 (13.9) | 7 (25.9) | 0.229 | 4 (12.1) | 10 (25.0) | 0.164 |

Abbreviations: MeVO, medium vessel occlusion; mRS, modified Rankin Scale; NIHSS, National Institute of Health Stroke Scale; ASPECTS, Alberta Stroke Program Early CT Score; IVT, intravenous thrombolysis; OTP, onset to puncture time; GTR, groin puncture to recanalization time; OTR, onset to recanalization time; LMC, leptomeningeal collaterals; EVT, endovascular treatment; IAT, Intra-arterial thrombolysis; MT, mechanical thrombectomy; mTICI, modified thrombolysis in cerebral infarction score; ENI, early neurological improvement; SICH, symptomatic intracranial hemorrhage; ICH, intracranial hemorrhage.

Bold values indicate statistical significance.

*Eight missing data.

※Seven missing data.

†One missing data.

‡Seventeen missing data.

§Six missing data.

Table S2. Unadjusted and adjusted model of efficacy and safety outcome in patients with primary versus secondary M2 occlusion by dominant/nondominant types

| Outcomes  Primary vs Secondary | Subgroups | Effect metric | Unadjusted model | | Adjusted model ^a^ | | P value for interaction ^a^ |
| --- | --- | --- | --- | --- | --- | --- | --- |
|  |  |  | Difference (95%CI) | P value | Difference (95%CI) | P value |  |
| 90-day mRS 0-2, n (%) | Dominant M2 | OR | 2.000 (0.712 to 5.619) | 0.188 | 1.856 (0.558 to 6.174) | 0.314 | 0.660 |
|  | Nondominant M2 |  | 1.750 (0.682 to 4.489) | 0.244 | 2.549 (0.814 to 7.987) | 0.108 |  |
| 90-day mRS 0-1, n (%) | Dominant M2 | OR | 1.342 (0.460 to 3.914) | 0.590 | 1.179 (0.343 to 4.050) | 0.794 | 0.860 |
|  | Nondominant M2 |  | 1.228 (0.479 to 3.148) | 0.669 | 1.363 (0.488 to 3.806) | 0.555 |  |
| 90-day mRS 0-3, n (%) | Dominant M2 | OR | 1.459 (0.532 to 4.004) | 0.463 | 1.808 (0.529 to 6.174) | 0.345 | 0.805 |
|  | Nondominant M2 |  | 1.505 (0.534 to 4.236) | 0.439 | 1.587 (0.512 to 4.918) | 0.423 |  |
| 90-day mRS, median (IQR)^b^ | Dominant M2 | cOR | 1.780 (0.731 to 4.338) | 0.204 | 1.720 (0.649 to 4.556) | 0.275 | 0.985 |
|  | Nondominant M2 |  | 1.477 (0.655 to 3.335) | 0.347 | 1.477 (0.625 to 3.489) | 0.374 |  |
| Early neurological improvement, n (%) | Dominant M2 | OR | 1.511 (0.522 to 4.378) | 0.447 | 1.363 (0.446 to 4.165) | 0.587 | 0.706 |
|  | Nondominant M2 |  | 1.061 (0.406 to 2.777) | 0.904 | 1.201 (0.430 to 3.355) | 0.726 |  |
| Change in NIHSS at 24 hours†, median (IQR)^c^ | Dominant M2 | GMR | 1.091 (0.971 to 1.225) | 0.143 | 1.097 (0.977 to 1.232) | 0.117 | 0.689 |
|  | Nondominant M2 |  | 1.058 (0.943 to 1.186) | 0.340 | 1.075 (0.959 to 1.205) | 0.126 |  |
| Change in NIHSS at 48 hours‡, median (IQR)^c^ | Dominant M2 | GMR | 1.090 (0.951 to 1.250) | 0.217 | 1.110 (0.964 to 1.279) | 0.145 | 0.620 |
|  | Nondominant M2 |  | 1.059 (0.934 to 1.200) | 0.372 | 1.075 (0.948 to 1.219) | 0.261 |  |
| Change in NIHSS at 7 days§, median (IQR)^c^ | Dominant M2 | GMR | 1.061 (0.896 to 1.257) | 0.491 | 1.110 (0.937 to 1.316) | 0.228 | 0.797 |
|  | Nondominant M2 |  | 1.060 (0.905 to 1.241) | 0.470 | 1.073 (0.922 to 1.249) | 0.364 |  |
| Change in NIHSS at discharge£, median (IQR)^c^ | Dominant M2 | GMR | 1.126 (0.932 to 1.359) | 0.218 | 1.149 (0.955 to 1.382) | 0.142 | 0.708 |
|  | Nondominant M2 |  | 1.072 (0.887 to 1.296) | 0.470 | 1.085 (0.900 to 1.308) | 0.391 |  |
| Symptomatic intracranial hemorrhage at 24 hours, n (%) | Dominant M2 | OR | 1.529 (0.131 to 17.797) | 0.734 | 1.083 (0.080 to 14.727) | 0.952 | 0.222 |
|  | Nondominant M2 |  | 0.264 (0.067 to 1.043) | 0.057 | 0.293 (0.068 to 1.268) | 0.101 |  |
| Any intracranial hemorrhage at 24 hours, n (%) | Dominant M2 | OR | 0.913 (0.303 to 2.750) | 0.872 | 0.956 (0.295 to 3.098) | 0.940 | 0.094 |
|  | Nondominant M2 |  | 0.269 (0.095 to 0.761) | **0.013** | 0.285 (0.091 to 0.888) | **0.030** |  |
| Mortality within 7 days, n (%)^d^ | Dominant M2 | HR | 0.286 (0.056 to 1.476) | 0.135 | 0.274 (0.048 to 1.564) | 0.145 | 0.327 |
|  | Nondominant M2 |  | 0.730 (0.174 to 3.055) | 0.666 | 0.716 (0.166 to 3.093) | 0.654 |  |
| Mortality within 90 days, n (%)^d^ | Dominant M2 | HR | 0.490 (0.155 to 1.545) | 0.224 | 0.321 (0.078 to 1.317) | 0.115 | 0.900 |
|  | Nondominant M2 |  | 0.475 (0.149 to 1.513) | 0.208 | 0.455 (0.139 to 1.496) | 0.195 |  |

Abbreviations: CI, confidence interval; OR, odds ratio; cOR, common odds ratio; GMR, geometric mean ratio; HR, hazard ratio; MeVO, medium vessel occlusion; mRS, modified Rankin Scale; NIHSS, National Institute of Health Stroke Scale.

Bold values indicate statistical significance.

^a^ Adjusted for key prognostic covariates (age, baseline NIHSS, pre-treatment with IVT and final mTICI).

^b^ Calculated with ordinal regression analysis.

^c^ The log (NIHSS+1) was analyzed using a generalized linear model.

^d^ Calculated with cox regression model.

†One missing data.

‡Eight missing data.

§Seventeen missing data.

£Six missing data.
